# Extracellular Monomeric Tau Is Internalized by Astrocytes

**DOI:** 10.3389/fnins.2019.00442

**Published:** 2019-05-01

**Authors:** Juan Ramón Perea, Esther López, José Carlos Díez-Ballesteros, Jesús Ávila, Félix Hernández, Marta Bolós

**Affiliations:** ^1^Department of Molecular Neuropathology, Centre for Molecular Biology “Severo Ochoa”, CSIC, Madrid, Spain; ^2^Network Center for Biomedical Research on Neurodegenerative Diseases, Madrid, Spain; ^3^Department of Biology and Chemistry, Alcalá University, Madrid, Spain

**Keywords:** Tau, tauopathies, astrocytes, heparan sulfate proteoglycans, internalization, propagation

## Abstract

Tau is a microtubule-associated protein that is expressed in neurons. However, in a group of neurodegenerative diseases named tauopathies – characterized by an increase in aggregated and/or hyperphosphorylated Tau – the protein accumulates inside other cells, such as astrocytes and microglia. Given that these glial cells do not produce Tau, its presence can be explained by internalization from the extracellular medium and consequent formation of Tau aggregates. Among internalization mechanisms, heparan sulfate proteoglycans (HSPGs) have been proposed to be responsible for fibrillary Tau uptake in various cell types. Here we studied whether monomeric Tau, which has been observed to be internalized by glial cells such as microglia, was also taken up by astrocytes. Although this Tau form was internalized from the extracellular medium by these cells, the mechanism of uptake was found to be independent of HSPGs.

## Introduction

Microtubule-associated protein Tau is expressed in neurons in the central nervous system (CNS) (Weingarten et al., 1975; Drubin and Kirschner, 1986). The abnormal aggregation and hyperphosphorylation of Tau accumulated within the cells is a common pathological hallmark of a group of neurodegenerative diseases named tauopathies (Spires-Jones et al., 2009; Medina et al., 2016). In this regard, the accumulation of this pathological and toxic Tau species in glial cells, specifically microglia and astrocytes, is described in several tauopathies (Buee and Delacourte, 1999; Kovacs et al., 2016; Ferrer et al., 2018). Given that glial cells do not contain endogenous Tau, the presence of this protein in this cell type can only be explained by its internalization from the extracellular milieu. In Alzheimer’s disease (AD), the most prevalent tauopathy, and Tau propagates following a stereotypical pattern (Braak and Braak, 1991). In the context of this disease, Tau is secreted to the medium and this extracellular form of the protein is taken up by a range of cells, including neurons, and glial cells (Gomez-Ramos et al., 2006; Calafate et al., 2015). Consequently, this internalization contributes to the spread of Tau from cell to cell (Holmes and Diamond, 2014; Hyman, 2014; Medina and Avila, 2014b).

Tau can be internalized by glial cells through several mechanisms (Leyns and Holtzman, 2017). In this regard, Tau aggregates are taken up by cultured cells, primary neurons, and brain through heparan sulfate proteoglycans (HSPGs) (Holmes et al., 2013; Stopschinski et al., 2018; Yamada and Hamaguchi, 2018). Furthermore, Tau fibril species are internalized in astrocytes via the lysosomal pathway (Martini-Stoica et al., 2018). These and other observations thus indicate that aggregated Tau is directly related to the progression of tauopathies. However, because monomeric Tau is considered less pathogenic, its internalization, and role in the progression of these diseases have received little attention. We have previously reported that monomeric Tau is internalized by microglia through a specific receptor, namely CX3CR1 (Bolos et al., 2017a). Furthermore, Tau triggers a pro-inflammatory profile through p38 MAPK in these cells, thereby contributing to the progression of neurodegeneration (Perea et al., 2018a). However, the uptake of monomeric Tau in astrocytes remains unknown.

To increase knowledge of Tau propagation mechanisms, and based on previous studies highlighting the role of HSPGs in the uptake of fibrillary Tau, here we addressed the involvement of HSPGs in the internalization of monomeric Tau in primary astrocyte cultures.

## Materials and Methods

### Animals

Wistar rats were used for the astrocyte primary culture experiments. The animals were obtained from the CBMSO (Centro de Biología Molecular Severo Ochoa) animal facility and were housed in a specific pathogen-free colony facility, in accordance with European Community Guidelines (directive 86/609/EEC), and handled following European and local animal care protocols (AEEC-CBMSO-62/14).

### Primary Cultures

Astrocytes were cultured from the cerebral cortices of P0-P3 rats. Cortices were dissected, stripped of meninges, and digested with 0.1% trypsin at 37°C in HBSS (Gibco, United States) medium for 10 min. Trypsinization was stopped by the addition of DMEM culture medium supplemented with 10% fetal bovine serum (Gibco, United States), 100 U/mL of penicillin, and 0.1 mg/mL of streptomycin (Gibco, United States). A single-cell suspension of the digested tissue was obtained by repeated pipetting. Cells were seeded into a 75-ml flask and cultured in medium at 37°C in humidified 5% CO_2_ – 95% air for 10 days. They were then seeded onto 12-well plates (2 × 10^5^ cells/well). Adherent cells were incubated for 72 h before being used for the experiments. For immunocytochemistry experiments, the cells were seeded onto 24-well plates (1 × 10^5^ cells/well) with coverslips.

### Human Monomeric Tau

The recombinant human Tau isoform, containing 2 N-terminal inserts, and 4 microtubule binding repeats (Avila et al., 2016), was isolated as previously described (Medina et al., 1995; Perea et al., 2018a). One mg/ml of purified Tau was labeled with sulfoindocyanine Cy5 dye (GE Healthcare, United Kingdom) as described previously (Gomez-Ramos et al., 2009) and following the manufacturer’s recommendations.

### Tau Internalization Assay

Primary astrocyte cultures were treated with either 1 μM of Tau-Cy5 or PBS-Cy5 for between 0 to 24 h. In the case of heparin or heparinase experiments, cultures were pre-treated with 20 μg/ml of heparin (Sigma, United States) or 10 mU/ml of heparinase III (Sigma, United States) for 3 and 2 h, respectively before treatment with Tau-Cy5 or PBS-Cy5, as described above. Afterward, the cells were washed three times with PBS in order to remove excess Tau attached to the membrane. They were then prepared for immunocytochemistry or immunoblotting analysis.

### Chinese Hamster Ovary (CHO) Cell Line

1.2 × 10^5^ cells/well were plated in a 24-well plate with coverslips. After 24 h, cells were treated with heparinase at 10 and 100 mU/ml or PBS, in the case of control conditions, for 2 h. Afterward, the cells were washed three times with PBS and prepared for immunocytochemistry as described below.

### Immunocytochemistry

After treatment, primary, culture and CHO cells were fixed in 4% paraformaldehyde in PBS for 15 min at room temperature (RT) and washed three times in PBS. Cells were then permeabilized with PBS containing 0.2% Triton X-100 for 10 min at RT and washed three times in PBS. After 30 min of blocking with 1% bovine serum albumin (BSA) in PBS containing 0,1% Tween20, the astrocytic cells were stained with rabbit anti-GFAP (1:1000, Synaptic Systems, Germany), and mouse anti-Cy5 (1:2000, Abcam, United Kingdom). CHO cells were stained with mouse anti-HSPG 10E4 (1:100, AMSbio, United Kingdom). All antibodies were diluted in blocking solution and incubated overnight at 4°C. The next day, cells were washed three times in PBS and then incubated for 1 h at RT with a donkey anti-rabbit IgG conjugated to Alexa Fluor 488 and donkey anti-mouse IgG conjugated to Alexa Fluor 647 (1:1000, Thermo Fisher Scientific, United States) for astrocytes, and donkey anti-mouse I gG conjugated to Alexa Fluor 488 (1:1000, Thermo Fisher Scientific, United States) for CHO cells. Finally, cells were washed three times in PBS, nuclei were labeled with DAPI (1:5000, Merck, Germany) for 10 min and rinsed with PBS.

### Confocal Image Acquisition and Image Analysis

Confocal stacks of images were obtained in a Zeiss LSM800 confocal microscope under 25× and 63× oil objectives. For the 25× objective the numerical aperture was 0.8, the interval between stacks: 670 nm and the size of the smallest pixel: 312 nm × 312 nm. In the case of 63× objective the numerical aperture was 1.4, the interval between stacks: 300 nm and the size of the smallest pixel: 132 nm × 132 nm. The quantification of the images was carried out on the maximum projection images using the 25× objective. Representative images were obtained with a 63× objective. Images were analyzed using ImageJ Version 1.50i (National Institutes of Health, United States).

Fluorescence intensity analysis: internalized Tau-Cy5 was measured by quantifying the levels of Cy5 staining inside cells. To this end, the contour of individual cells was drawn in the GFAP channel and the total area was measured. Images were subjected to an invariant threshold and the Cy5^+^ area above the threshold was measured and then divided by the selected area of the cell. To determine the amount of HSPGs in CHO cells, images were subjected to an invariant threshold and the HSPG^+^ area above the threshold was measured and then divided by the selected area of the cell. Data relative to fluorescence intensity are thus presented as % of positive area. 150 astrocytes or 50 CHO cells were analyzed for each experimental condition and time point.

### Immunoblotting

The levels of Tau and β-actin in primary astrocyte cultures were determined by western blotting. After treatments, cells were lysed in RIPA buffer [50 mM Tris–HCl, pH 7.4, 150 mM NaCl, 1% Triton X-100; 0.5% Sodium Deoxycholate, 0.1% SDS, and protease inhibitor mixture (Roche, Switzerland)]. Proteins were then separated on 10% sodium dodecyl sulfate-polyacrylamide gels in reducing conditions before being transferred electrophoretically onto nitrocellulose membranes (GE healthcare, United Kingdom). Membranes were blocked for 2 h with 5% in BSA in 50 mM of Tris–buffered saline, pH 8, containing 0.05% of Tween 20 (TBS-Tween). They were then incubated overnight at 4°C with the following antibodies: mouse anti-Tau5 antibody (1:1000, Merck, Germany); mouse anti-Cy5 antibody (1:1000, Abcam, United Kingdom); or mouse anti-β-actin antibody (1:1000, Sigma, United States). Protein expression was detected using HRP-conjugated secondary antibodies (1:2000, Agilent, United States). For quantification of immunoreactivity, images of blots were analyzed using ImageJ Version 1.50i (National Institutes of Health, United States). All results were obtained in at least three independent experiments.

### Statistical Analysis

Statistical analysis was performed with GraphPad Prism software, Version 7.0a (United States). Data were tested by two-way analysis of variance. Post-hoc comparisons were analyzed using Holm-Sidak’s or Tukey’s test. Differences were considered statistically significant when the probability, *p*, of the null hypothesis was ≤0.05. Data are presented as the means ± SE.

## Results

### Astrocytes Internalized Monomeric Tau From the Extracellular Medium

**FIGURE 1:**
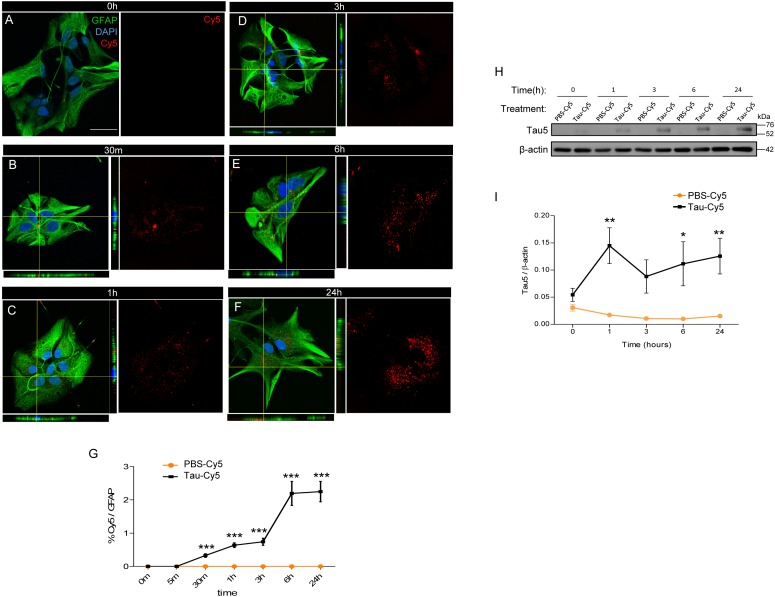
Astrocytes internalized extracellular Tau *in vitro*. Astrocytes derived from primary cultures were incubated with Tau-Cy5 at different time points **(A**–**G)**. **(A**–**F)** show representative immunofluorescence images of cells stained for GFAP (green) and Tau (red). Representative images of times 0 h **(A)**, 30 min **(B)**, 1 h **(C)**, 3 h **(D)**, 6 h **(E)**, and 24 h **(F)** are shown. **(G)** shows quantification of results from immunofluorescence analysis showing % Cy5/GFAP fluorescence intensity present in cells treated with Tau-Cy5 relative to the control PBS-Cy5. **(H)** western blot and **(I)** quantification, of Tau in cell lysates from 0 to 24 h. Means and SE, PBS 0 h = 0.03 ± 0.006; Tau 0 h = 0.05 ± 0.01; PBS 1 h = 0.02 ± 0.002; Tau 1 h = 0.14 ± 0.03; PBS 3 h = 0.01 ± 0.002; Tau 3 h = 0.09 ± 0.03; PBS 6 h = 0.01 ± 0.003; Tau 6 h = 0.11 ± 0.04; PBS 24 h = 0.01 ± 0.002; and Tau 24 h = 0.12 ± 0.03. An *N* = 5 independent experiments were performed. Bars show means ± SE. ^∗^*p* ≤ 0.05, ^∗∗^*p* ≤ 0.01, and ^∗∗∗^*p* ≤ 0.001. Scale bar, 50 μm.

The internalization of Tau fibrils into astrocytes has been described (Martini-Stoica et al., 2018). However, the uptake of monomeric Tau, which has a key role in tauopathies (Fox et al., 2011; Gerson et al., 2014; Bolos et al., 2017b), by these cells remains unknown. In this regard, here we studied the internalization of human monomeric Tau (Tau) in astrocyte primary cultures at time points ranging from 0 to 24 h (Figure 1). To facilitate the detection of Tau inside the cells by immunocytochemistry, the protein was labeled with Cy5 (Tau-Cy5), as previously described (Bolos et al., 2015). PBS-Cy5 was used as a control. The amount of Cy5 (in red) internalized (GFAP, green color) was measured at different times (Figure 1A–G). At time 0 h, no Cy5 was detected inside the cells (Figure 1A). However, as early as 30 min, Cy5 staining, denoting Tau presence, was observed and increased over time until 24 h, when seems to reach a plateau (Figure 1B–G).

To corroborate that the Tau detected in the astrocytes derived from the extracellular medium rather the cells themselves, the cells were treated with Tau-Cy5, or control (PBS-Cy5). The amount of Tau internalized was then analyzed in cell lysates by western blot using Tau5, a Tau antibody (Figure 1H). The quantification is shown in Figure 1F. The presence of Tau protein was not detected at early times, thereby supporting the notion that the source of Tau is extracellular (Figure 1H). However, after 1 h and until 24 h, Tau increased inside the cells compared to controls (Figure 1H,I).

These results confirm that Tau in astrocytes derives from the extracellular medium and that its internalization increases over time.

### Heparan Sulfate Proteoglycans (HSPGs) Are Not Involved in the Internalization of Monomeric Tau

**FIGURE 2:**
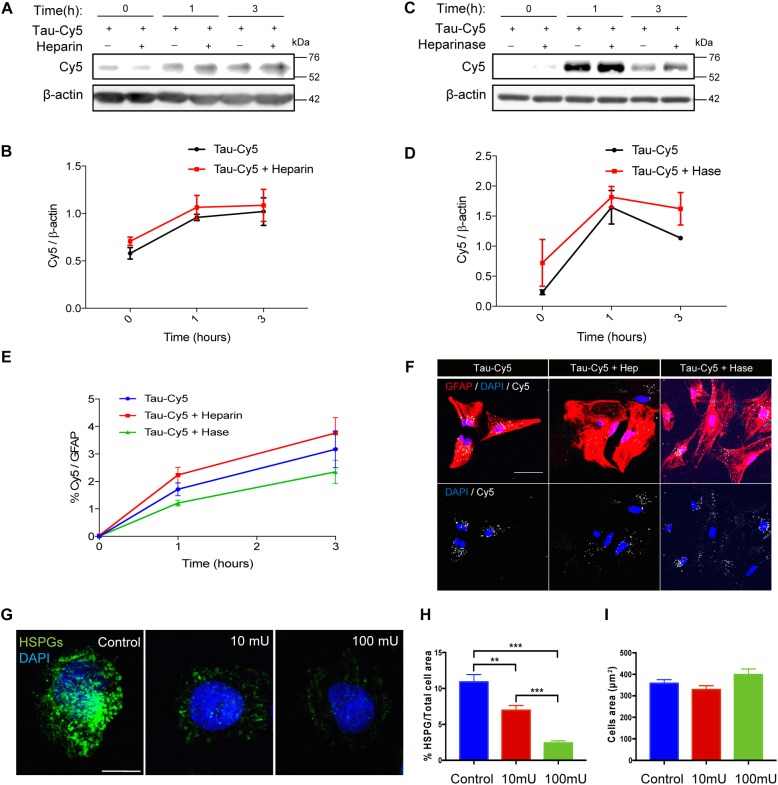
Internalization of monomeric Tau in astrocytes is not through heparan sulfate proteoglycans. Representative western blot **(A)** and quantification **(B)** of the time course of Tau-Cy5 internalization from 0 to 3 h in the presence or absence of heparin. Cells were treated with Tau-Cy5 (control, T) or Tau-Cy5 + heparin (T+Hep) for different times, and Cy5 was analyzed in cell lysates. Note that the internalization of Tau did not change in the presence of heparin. Means and SE, T 0 h = 0.58 ± 0.06; T+Hep 0 h = 0.71 ± 0.04; T 1 h = 0.96 ± 0.03; T+Hep 1 h = 1.06 ± 0.13; T 3 h = 1.02 ± 0.15; and T+Hep 3 h = 1.09 ± 0.17. Representative western blot **(C)** and quantification **(D)** of Tau-Cy5 with (T+Hase) or without heparinase treatment (T) from 0 to 3 h. Means and SE: T 0 h = 0.23 ± 0.04; T+Hase 0 h = 0.72 ± 0.4; T 1 h = 1.65 ± 0.28; T+Hase 1 h = 1.82 ± 0.17; T 3 h = 1.13 ± 0.03; and T+Hase 3 h = 1.62 ± 0.27. Note that the internalization of Tau did not change after the treatment with heparinase. Quantification of Tau-Cy5 internalization measured by immunocytochemistry **(E)** and some representative images of 1 h time point after treatment **(F)** confirm the results obtained by western blot. Heparinase treatment reduces HSPGs **(G**–**I)**. Representative images of CHO cells treated with 0, 10, or 100 mU/ml of heparinase **(G)** and quantification of HSPGs **(H)**. Means and SE, control = 10.9 ± 1.02; heparinase 10 mU = 6.9 ± 0.67; and heparinase 100 mU = 2.4 ± 0.29. Quantification of the total area of the cells **(I)** support that heparinase treatment reduces the presence of HSPGs in the cells. An *N* = 3 independent experiments were performed. Bars show means ± SE. ^∗∗^*p* ≤ 0.01 and ^∗∗∗^*p* ≤ 0.001. Scale bar, 50 μm **(F)** and 10 μm **(G)**.

The internalization of Tau in aggregate and fibrillary forms through HSPGs has been studied using various cell models (Holmes et al., 2013; Martini-Stoica et al., 2018). However, the implication of these structures in the internalization of monomeric Tau has been addressed only in neurons (Katsinelos et al., 2018; Rauch et al., 2018). Hence, following the same protocol described in previous studies (Ihse et al., 2017), here we studied the uptake of monomeric Tau by astrocytes through HSPGs (Figure 2). Using primary cultures of astrocytes, the internalization of Tau was analyzed at different times in the presence or absence of heparin (Figure 2A,B). Heparin can be used to competitively inhibit binding to HSPGs and prevent Tau uptake via these structures (Holmes et al., 2013). After 1 and 3 h of heparin treatment, Tau protein was detected inside the cells independently of the presence of heparin (Figure 2A,B). In addition, the amount of Tau in primary cultures treated with heparinase, which removes HSPGs (Figure 2C,D), was measured. After 1 h, Tau was found inside the astrocytes that were treated with heparinase, in the same way as those not treated. These results were confirmed using immunocytochemistry approach (Figure 2E,F). The quantification (Figure 2E) and the representative images after 1 h of Tau-Cy5 treatment with or without heparin and heparinase (Figure 2F), confirm that the amount of Tau inside the cells were the same. In order to be sure that HSPGs were removed properly, CHO cells were treated with 0 (control), 10, or 100 mU/ml of heparinase for 2 h (Figure 2G,H). The representative images (Figure 2G) and quantification (Figure 2H) confirm that the amount of HSPGs was reduced after heparinase treatment. As a control, the total area of the cells was measured after the heparinase treatment and no changes were observed (Figure 2I). These results therefore suggest that monomeric Tau is internalized by these cells through a mechanism that is not mediated by HSPGs.

## Discussion

Tauopathies are a group of disorders characterized by the abnormal accumulation of the microtubule-associated protein Tau (Goedert and Spillantini, 2011). In tauopathies such as AD, intracellular Tau forms filamentous of hyperphosphorylated and aggregated protein inside the cells that is associated with the progression of neurodegeneration (Ballatore et al., 2007; Goedert and Spillantini, 2011; Arendt et al., 2016; Medina et al., 2016). In these diseases, Tau inclusions have also been reported in glial cells, such as microglia (Odawara et al., 1995; Bolos et al., 2015; Leyns and Holtzman, 2017) and astrocytes (Kovacs et al., 2016; Ferrer et al., 2018), despite their lack of Tau expression. These observations could be explained by the internalization of Tau previously released into the extracellular space through physiological and pathological mechanisms (Simon et al., 2012; Pooler et al., 2013; Perez et al., 2016) and its posterior secretion again by neighboring cells (Perea et al., 2018b). The transfer of Tau between brain cell populations (Holmes and Diamond, 2014; Medina and Avila, 2014a; Calafate et al., 2015; Maphis et al., 2015) leads to its propagation throughout the CNS.

Although fibrillary and aggregated Tau, which are formed by hyperphosphorylated Tau, are considered a histopathological hallmark of tauopathies (Selkoe et al., 1982), monomeric forms of the protein are also involved in toxicity and the spread of the disease (Gomez-Ramos et al., 2006; Cowan and Mudher, 2013; Gerson et al., 2014; Bolos et al., 2017b; Perea et al., 2018a). In this regard, we previously reported the internalization of extracellular monomeric Tau by microglia (Bolos et al., 2015), and in the present work we confirmed the same event in astrocyte primary cultures. These results support the notion that glial cells contribute to the cell-to-cell propagation of Tau (Leyns and Holtzman, 2017; Perea et al., 2018b).

Several mechanisms of extracellular Tau internalization in neurons (Wang et al., 2017; Katsinelos et al., 2018) and glial cells have been described (Luo et al., 2015; Bolos et al., 2017a). Among them, HSPGs have been shown to be involved in the uptake of fibrillary and aggregated Tau (Holmes et al., 2013; Martini-Stoica et al., 2018; Rauch et al., 2018). However, here we have demonstrated that the uptake of monomeric Tau in astrocytes is independent of HSPGs. Both in the presence of heparin, which competes with Tau for binding to HSPGs, as well as after HSPGs removal using heparinase, the amount of internalized Tau in astrocytes was the same. This observation implies that another internalization mechanism is responsible for the uptake of monomeric Tau species by astrocytes. These results are in agreement with similar findings obtained in a recent study performed with α-synuclein (Ihse et al., 2017). Those authors demonstrated that only aggregated α-synuclein, and not other species of the protein, was internalized into a range of glial cells through HSPGs.

It appears that the mechanism by which fibrillary and/or aggregated Tau enters cells differs from that used by the monomeric Tau form. In this regard, monomeric Tau is internalized by muscarinic receptors M1 and M3 (Gomez-Ramos et al., 2006) in neurons; by CX3CR1 receptors in microglia (Bolos et al., 2017a); and by a non-HSPG-mediated mechanism in astrocytes. In contrast, the aggregated and fibrillary form of Tau is taken up by neurons via the HSPG pathway. These differences could be relevant for the design of potential drugs that target receptors and have the capacity to protect against the propagation of Tau in neurodegenerative diseases.

In summary, here we have shown that extracellular monomeric Tau is taken up by astrocytes. However, contrary to what we expected, this internalization is not HSPG-dependent. The mechanism by which monomeric Tau enters astrocytes is still unknown and deserves further attention.

## Ethics Statement

This study was carried out in accordance with European Community Guidelines (directive 86/609/EEC) and handled following European and local animal care protocols. Animal experiments received the approval of the CBMSO’s Ethics Committee and the National Ethics Committee (AEEC-CBMSO-62/14).

## Author Contributions

JD-B, JÁ, FH, and MB conceived and designed the study. JP, EL, and MB collected the data. JP and MB analyzed the data. MB wrote the first draft of the manuscript. All the authors read and approved the final manuscript.

## Conflict of Interest Statement

The authors declare that the research was conducted in the absence of any commercial or financial relationships that could be construed as a potential conflict of interest.
